# Aspirin for Prevention of Preeclampsia in Lupus Pregnancy

**DOI:** 10.1155/2014/920467

**Published:** 2014-03-20

**Authors:** Amelie M. Schramm, Megan E. B. Clowse

**Affiliations:** ^1^Department for Internal Medicine 3 and Institute for Clinical Immunology, Friedrich-Alexander-University Erlangen-Nuremberg, Erlangen, Germany; ^2^Department of Medicine, Duke University Medical Center, P.O. Box 3535, Trent Drive, Durham, NC 27710, USA

## Abstract

Preeclampsia, the onset of hypertension and proteinuria during pregnancy, is a common medical disorder with high maternal and fetal mortality and morbidity. The underlying pathology remains poorly understood and includes inflammation, endothelial dysfunction, and an unbalanced thromboxane A_2_/prostacyclin ratio. For women with systemic lupus erythematosus (SLE), particularly those with preexisting renal disease or with active lupus, the risk of developing preeclampsia is up to 14% higher than it is among healthy individuals. The mechanism is still unknown and the data for preventing preeclampsia in lupus pregnancies are rare. Modulating the impaired thromboxane A_2_/prostacyclin ratio by administration of low-dose aspirin appears to be the current best option for the prevention of preeclampsia. After providing an overview of the pathogenesis of preeclampsia, preeclampsia in lupus pregnancies, and previous trials for prevention of preeclampsia with aspirin treatment, we recommend low-dose aspirin administration for all lupus patients starting prior to 16 weeks of gestation. Patients with SLE and antiphospholipid syndrome should receive treatment with heparin and low-dose aspirin during pregnancy.

## 1. Introduction

Preeclampsia is defined by an increase in blood pressure (>140/90) and proteinuria (>300 mg/24 hr) in the latter half of pregnancy. While modern obstetrical management has made it less dangerous than before, it is still associated with an increased rate of maternal and fetal mortality and is an important cause for preterm birth [1]. It is difficult to identify pregnancies at particularly high risk for preeclampsia before it presents clinically; however, there are several known risk factors. These include first birth, a first birth with a new father, prior hypertension or renal disease, diabetes, and prior preeclampsia. Both systemic lupus erythematosus (SLE) and antiphospholipid syndrome (APS) put pregnancies at high risk for this complication. Uterine Doppler studies can identify pregnancies with particularly high uterine artery pressures, which may be an indicator of early placental changes that lead to preeclampsia [1]. The goal of this paper is to provide insight into the mechanisms through which SLE and APS contribute to preeclampsia and the potential role that low-dose aspirin may play in mitigating this risk.

## 2. Pathogenesis

### 2.1. Pathophysiology of Preeclampsia

As a common medical disorder during pregnancy, preeclampsia causes hypertension (systolic blood pressure >140 mmHg or diastolic blood pressure >90 mmHg), proteinuria (>300 mg/24 h), and in rare cases additional symptoms like hyperreflexia, seizures (eclampsia), acute renal failure, pulmonary complications, the triad of hemolysis, elevated liver enzymes, and low platelets (HELLP syndrome).

**Figure 1:**
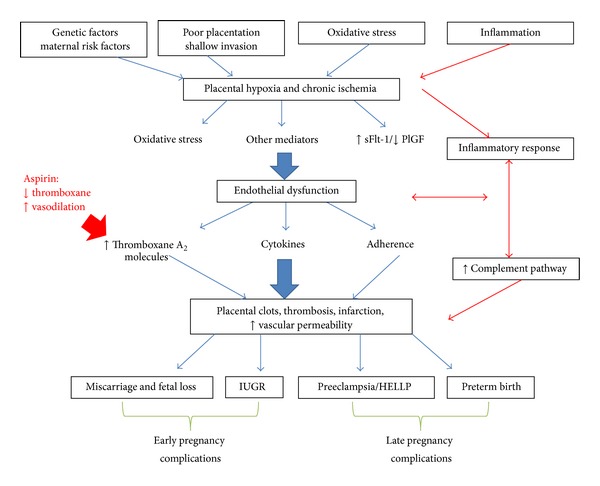
Mechanisms of preeclampsia.

The disease typically starts after 20 weeks of gestation and is an important factor for maternal and fetal mortality and morbidity. In the United States, around 5–8% of pregnant women are affected [2]. The only definitive treatment is delivery of the baby. While preeclampsia's underlying pathology remains poorly understood, the onset of this condition involves shallow trophoblast invasion with poor placentation, inflammation, dysregulation of angiogenic factors, and ischemia, all of which lead to the central mechanism of endothelial dysfunction. Endothelial dysfunction causes, among other things, activation of platelets, a rise in thromboxane levels, and an ensuing clotting cascade (Figure 1).

#### 2.1.1. Poor Placental Vascular Remodeling

Implantation and placental development happen in the first trimester of pregnancy. Fetal cytotrophoblasts invade the maternal spiral arteries and convert these high-resistance, muscular arteries to high-capacitance, elastic vessels. Insufficient spiral artery transformation is strongly associated with the pathology of severe preeclampsia. In preeclamptic placentas, shallow trophoblast invasion prevents the necessary vascular remodeling, which leads to decreased perfusion, hypoxia, and chronic placental ischemia [3].

#### 2.1.2. Imbalance of Angiogenic Factors

Chronic ischemia caused by poor vascularization is associated with the placental production of angiogenic factors like vascular endothelial growth factor (VEGF), placental growth factor (PlGF), and soluble fms-like tyrosine kinase-1 (sFlt-1). VEGF encourages the growth of blood vessels, supports proper function of endothelial cells, and stimulates NO production in vascular walls [4, 5]. SFlt-1 is a naturally occurring VEGF antagonist which binds free VEGF and occupies the VEGF receptor [6]. Experiments and animal models suggest that VEGF and sFlt-1 may play a role in the pathogenesis of preeclampsia [7]. During healthy pregnancies, levels of placental growth factor (PlGF) increase in the first and second trimesters and fall in the third trimester. The level of antiangiogenic factor sFlt-1 usually remains steady during the first part of pregnancy and rises during the last trimester. In blood samples of patients with preeclampsia, however, lower levels of PlGF throughout gestation and increased levels of sFlt-1 levels at 26 and 29 weeks of gestation are detectable [8–10]. An elevated sFlt-1/PlGF ratio during the second trimester, but not the first trimester, can help detect preeclampsia even before the presentation of clinical symptoms [11–13]. Increased levels of sFlt-1 also can be found in lupus patients with preeclampsia [14].

#### 2.1.3. Inflammation

Systemic maternal inflammation is also involved in the pathogenesis of preeclampsia. Levels of circulating proinflammatory mediators like IL-6, IL-8, TNF-*α*, and monocyte chemoattractant protein 1 (MCP-1) are markedly higher in preeclamptic pregnancies than in healthy pregnancies [19]. Noninfectious leukocyte infiltration of the villi (fetal) and the decidua (maternal interface) was found in preeclamptic placentas, along with an uncontrolled, increased activation of the complement system, with elevated levels of complement-activation factor Bb. Activation of the complement system is commonly seen as an important mechanism linking inflammation and coagulation [20]. Inflammation may therefore be a trigger for the development of preeclampsia in patients with SLE [21].

#### 2.1.4. Thromboxane

Poor placental perfusion leads to activation of platelets and the clotting cascade, resulting in an imbalance among vasoactive prostaglandins. The ratio between the prostaglandins thromboxane and prostacyclin modulates vascular blood flow, with thromboxane A_2_ acting as a vasoconstrictor and promoting platelet aggregation, while prostacyclin acts as a vasodilator and inhibits aggregation. Increased thromboxane and reduced prostacyclin levels are associated with infarction and thrombotic vasculopathy, which are well-known features in preeclamptic placentas [22].

**Figure 2:**
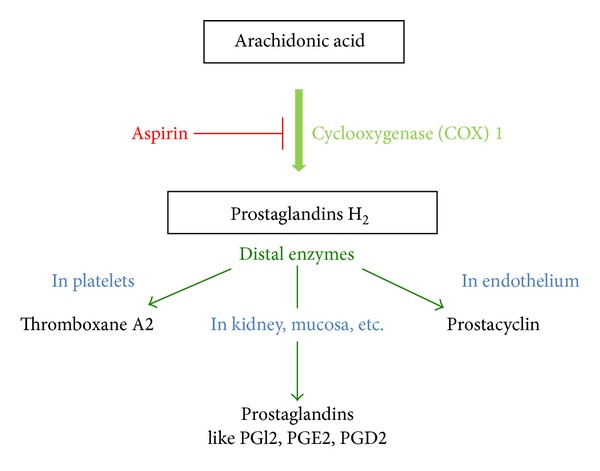
Interaction of aspirin with COX 1.

The constitutive enzyme cyclooxygenase 1 produces thromboxane A_2_ in platelets and primarily prostacyclin in endothelial cells. Aspirin, a common irreversible inhibitor of cyclooxygenases, acts particularly in platelets. The impact of aspirin use in preeclampsia prevention is shown by data which demonstrate an aspirin-induced decrease of thromboxane concentration and mediation of the unbalanced thromboxane A_2_/prostacyclin ratio [23] (Figure 2). Aspirin thus improves placental blood flow and minimizes risk of placental thrombosis, which serves as the rationale for administering prophylactic low-dose aspirin for prevention of preeclampsia.

### 2.2. Pregnancy in Patients with Systemic Lupus Erythematosus

Systemic lupus erythematosus (SLE) is a pervasive autoimmune disease which can affect nearly every organ and tissue in the body with an extremely broad variability in severity. Women with SLE are often diagnosed in their childbearing years [24]. As SLE usually has no influence on female fertility, pregnancies are common among these women [25].

Maternal and fetal risk for serious medical and pregnancy complications is significantly higher for women with SLE than for healthy women. National analysis showed a 20-fold higher risk for maternal mortality among lupus patients, who are also at increased risk for preterm labor (OR 2.4), Cesarean section (OR 1.7), and fetal growth restriction (OR 2.6) [26]. Compared to healthy individuals, women with SLE, especially those with preexisting renal disease or with active SLE before and during pregnancy, have a higher risk for developing preeclampsia. Up to 30% of all lupus pregnancies are complicated by preeclampsia [27, 28].

While active lupus is the main predictor for pregnancy complications, other identified risk factors are strongly associated with preeclampsia, including preexisting hypertension, antiphospholipid syndrome (APS), obesity, positive anti-double-stranded DNA antibodies (dsDNA) or anti-ribonucleoprotein (RNP) antibodies, low complement [2], and thrombocytopenia at onset of pregnancy [29]. Thrombocytopenia that occurs in lupus pregnancy before 15 weeks of gestation is usually due to SLE activity (platelet-specific antibodies) or APS. After 25 weeks, low platelet counts are more commonly caused by preeclampsia/HELLP syndrome [27].

Differentiating between lupus activity and pregnancy-related complications presents a major challenge in the management of SLE pregnancies. In both SLE and preeclampsia, for example, women develop worsening proteinuria with hypertension and edema. Yet it remains essential to accurately determine whether symptoms are due to lupus or to preeclampsia, given the different treatments required for preeclampsia (delivery) and SLE (immunosuppression) [2]. Decreased levels of complement and active urine sediment could suggest lupus nephritis, whereas elevated serum uric acid and low urine calcium are more typical for preeclampsia. Additionally, the presence of concomitant lupus symptoms—like arthritis, serositis, skin lesions, or rising levels of dsDNA-antibodies—could point to SLE activity.

While women who experience increased SLE activity during pregnancy are the most at risk, even those with quiescent lupus are more likely to develop preeclampsia than healthy women. This suggests that SLE and preeclampsia may share a common underlying pathology.

#### 2.2.1. Endothelial Dysfunction

Chronic autoimmune diseases with systemic inflammation lead to an increased risk for cardiovascular disease (CVD), with atherosclerosis and vascular alterations through changes in vascular adhesion molecules, increased transendothelial permeability, impaired antithrombotic properties, and reduced production of vasodilators and thrombomodulin expression. Thrombomodulin limits aggregation of platelets and activation of the complement pathway. The presence of proinflammatory cytokines, however, lowers thrombomodulin and increases levels of the procoagulant tissue factor [30].

Impaired endothelial repair is also linked to endothelial dysfunction in SLE. While patients with SLE have similar numbers of endothelial progenitor cells compared to healthy people, these cells exhibit impaired migratory and adhesive properties [31]. We suggest that the endothelial dysfunction inherent in SLE may contribute to the risk of preeclampsia in this population.

#### 2.2.2. Inflammation

During pregnancy, T-cells play an important role in modulating the maternal immune system as it adapts to a semiallogeneic fetus [32]. Fewer regulatory T-cells (Treg) and increased T helper-17 cell (Th17) activity have been found in women with preeclampsia. Similar changes are common in active SLE. One study of SLE pregnancies, for example, documented particularly low levels of Treg cells in the context of preeclampsia [33–36]. We suggest that the immune dysregulation seen in SLE may contribute to the risk of preeclampsia. Reduced systemic inflammation and normalized T-cell activity during lupus quiescence may lead to a reduction of pregnancy complications in the absence of lupus activity.

### 2.3. Antiphospholipid Syndrome

Antiphospholipid syndrome (APS) is a prothrombotic disorder which can cause thrombosis, embolism, or stroke and is highly associated with pregnancy complications like miscarriage and preeclampsia. The antibodies of APS are circulating anti-phospholipid antibodies (aPL), which are characterized as anti-cardiolipin antibodies (aCL), lupus-anticoagulant (LAC), and *β*2-glycoprotein antibodies (anti-*β*2-GPI). Patients are diagnosed by the combination of detectable antibodies and clinical findings (including thrombosis, embolism, and stroke) or obstetrical failures (including three sequential early pregnancy losses, a second- or third-trimester loss, or severe early preeclampsia).

#### 2.3.1. APS and Pregnancy

The risk for preeclampsia is more than ninefold higher in APS patients than in healthy women [37]. The pregnancy complications of APS appear to result from the interaction of prothrombotic factors, inflammation, and trophoblast pathologies. Phospholipid-binding proteins—annexin V, protein C, prothrombin, or anti-*β*2-GPI—are antigen targets for aPL. Whereas most human cells translocate *β*2-glycoprotein on their surface only during apoptosis or pathological conditions, trophoblasts continually present *β*2-GPI on their cell membranes. This could explain *β*2-GPI placental tropism and placenta-related pregnancy complications in women with APS [38].

Clots, thrombosis, and placental infarction due to the presence of antibodies and the following platelet activation are common in APS placentas. While aPL does not react with resting endothelial cells, Chen et al. found that a triggering event such as phagocytosis of necrotic trophoblastic debris allows aPL to influence endothelial cells for a prolonged period. After a trigger event, aPL can maintain activation of endothelial cells even without the further presence of necrotic trophoblastic debris. Chronic activation of the endothelium leads to an imbalance between thromboxane A_2_ and prostacyclin [39]. Correction of this imbalance by low-dose aspirin may explain the benefit of aspirin administration in APS pregnancy.

Pregnant women with anti-phospholipid antibodies are at risk for catastrophic APS (CAPS), which presents with rapid evolution of thrombosis, often microthrombi, in 3 or more organs. CAPS is deadly: up to half of women who develop this in pregnancy die, as do half of the infants. The role that aspirin might play in the prevention or treatment of CAPS is unclear, but aggressive anticoagulation, plasmapheresis, and immunosuppression are all suggested therapies. CAPS may present concurrently with preeclampsia and/or HELLP (hemolysis, elevated liver enzymes, and low platelets) syndrome [40].

Treatment guidelines suggest combining therapies of low-dose aspirin and heparin for all APS patients during pregnancy (Table 1). Depending on the patient's history of thrombosis, miscarriage, or other pregnancy complications, the medication may be extended to anticoagulation with full therapeutic dose [41, 42].

Studies have documented a decrease in pregnancy loss among patients with APS who receive heparin treatment, but heparin does not appear to be as effective in preventing late pregnancy complications such as preeclampsia [42]. In addition to its anticoagulant properties, heparin can interrupt the interaction between aPL and *β*2-GPI, inhibit the complement pathway, and provide supplemental anti-inflammatory effects, but it does not influence trophoblast migration or placentation. This may explain heparin's lack of significant benefit for prevention of late pregnancy complications like preeclampsia, despite its effective prevention of early pregnancy problems in APS patients [38]. Aspirin, on the other hand, may play a role in preventing late pregnancy complications.

## 3. Clinical Trials of Low-Dose Aspirin Administration for Prevention of Preeclampsia

Until recently, preeclampsia has been resistant to preventive treatment. Low-dose aspirin, however, has shown beneficial effects in a wide range of clinical trials for prevention of placenta-associated pregnancy complications. While the definition of high risk for preeclampsia has not been entirely consistent between studies, patients identified as high risk typically have a history of preeclampsia or fetal growth restriction, abnormal uterine artery Doppler, essential hypertension, obesity, and/or diabetes mellitus. A few studies also included women with underlying vascular disorders or autoimmune diseases, but the number of patients studied with rheumatologic disease is very small.

Two meta-analyses demonstrated beneficial effects of aspirin for prevention of preeclampsia: among women at high risk for this complication, antiplatelet treatment reduced the risk by 17–21% (Table 2) [15, 16]. In Trivedi's analysis, the incidence of preeclampsia in the high-risk group was 10.7% with low-dose aspirin administration and 12.5% with placebo administration (RR 0.79, *P* = 0.02). The low-risk group, however, showed no significant differences in preeclampsia incidence with aspirin (4.3%) and with placebo (4.4%, RR 0.86, *P* = 0.35). These meta-analyses included fairly heterogeneous studies, with low-dose aspirin initiation ranging from 7 to 32 weeks of gestation.

More robust findings emerged from two meta-analyses that were restricted to studies in which low-dose aspirin was started prior to 16 weeks of gestation. One analysis found that while low-dose aspirin introduced <16 weeks of gestation decreased the risk for severe preeclampsia, perinatal death, and fetal growth restriction, low-dose aspirin initiation after 16 weeks of gestation did not provide this protective effect [17]. Villa et al. demonstrated significantly reduced risk for preeclampsia (RR 0.6, CI 0.27–0.83) and severe preeclampsia (RR 0.3, CI 0.11–0.69) with low-dose aspirin administration in women with abnormal uterine artery flow [18]. Roberge et al. found that low-dose aspirin administration resulted in an 89% risk reduction for preterm preeclampsia but did not decrease risk for term preeclampsia. Based on this data, it appears that early initiation of low-dose aspirin is important and that it may be most effective in preventing preterm and severe preeclampsia [17].

The range of aspirin dosages with positive effects appears to be quite flexible. Across studies, the administered low-dose aspirin dose was between 40 and 160 mg/day, yet there was no difference in potency [16]. Previous trials also investigated the maternal and neonatal outcomes of pregnancies exposed to low-dose aspirin, and treatment appears to be safe for both mother and newborn [15, 43]. Compared to women in the control groups, for example, pregnant women treated with low-dose aspirin experienced no significant difference in risk of maternal or neonatal bleeding [16]. Case-controlled data also showed no increased risk of congenital abnormalities [44]. And, unlike high-dose NSAIDs, low-dose aspirin does not appear to increase the risk for ductus arteriosus closure* in utero* [45]. Analysis for preconceptional low-dose aspirin administration for prevention of preeclampsia after* in vitro* fertilization (IVF) found no significant reduction of hypertensive pregnancy complications compared to placebo group [22].

## 4. Conclusion: Impact of Anticoagulation for Prevention of Preeclampsia in Lupus Patients

The task of lowering the risk for preeclampsia in women with SLE is challenging. Maintaining SLE quiescence may reduce this risk by minimizing the impact of chronic inflammation, but endothelial dysfunction due to systemic lupus is not currently amenable to treatment. Aspirin, however, can interfere with the subsequent pathological process of a vasoconstrictive, procoagulant, and platelet-activating state and may prevent preeclampsia by modulating the thromboxane A_2_/prostacyclin ratio to optimize placental blood flow and prevent placental thrombosis.

While aspirin will not eliminate all cases of preeclampsia, it is currently the best and safest available drug for influencing the pathogenesis and clinical presentation of preeclampsia. Although there is no trial evidence for the use of low-dose aspirin to prevent preeclampsia in SLE, aspirin could lower the risk for lupus patients to an extent comparable to the risk reduction demonstrated in trials assessing low-dose aspirin treatment for other high-risk groups. Therefore, we would expect aspirin treatment to offer a risk reduction of up to 20% for preeclampsia development in lupus patients, suggesting the possibility of lowering preeclampsia incidence from 15% in all lupus pregnancies to around 12% in lupus patients with low-dose aspirin treatment.

### 4.1. Preeclampsia Prophylaxis in Lupus Pregnancies

Based on the pathogenic role of thromboxane in placental perfusion and the higher incidence of preeclampsia in SLE pregnancy, we recommend low-dose aspirin administration for all pregnant women with SLE, with therapy being initiated prior to 16 weeks of gestation and continuing throughout pregnancy. Women with SLE and APS should continue aspirin treatment as a preeclampsia prophylaxis and add heparin or LMWH. For pregnant women without a history of thrombosis, lower prophylactic dosing of heparin or LMWH is appropriate, but for pregnant women with a history of thrombosis, heparin administration should be increased to a full antithrombotic dose.

A future clinical trial of the use of aspirin as preeclampsia prevention in SLE should be performed to generate more exact recommendations.

## Figures and Tables

**Table 1:** Treatment of anti-phospholipid antibodies in pregnancy.

Clinical presentation	Suggested treatment
Patients with aPL and no history of thrombosis and no series of fetal loss or early delivery due to preeclampsia or placental insufficiency	Addition of low-dose aspirin throughout pregnancy

Patients with APS and no history of thrombosis but with previous history of stillbirth, recurrent fetal loss, or other APS-associated pregnancy complications	Heparin or LMWH (usual prophylactic dose) during pregnancy and 6 weeks postpartum Low-dose aspirin throughout pregnancy

Patients with APS and prior history of thrombosis or embolism	Heparin or LMWH (usual therapeutic dose) during pregnancy and 6 weeks postpartum followed by optional conversion on warfarin Low-dose aspirin throughout pregnancy

**Table 2:** Data from meta-analyses of aspirin for preeclampsia prevention.

Meta-analysis (first author)	Onset of treatment	Inclusion criteria	Intervention	Methods	Results
Duley et al. Cochrane Review 2007 [15]	Before and after 20 weeks of gestation	*High risk criteria*: Previous severe preeclampsia Chronic hypertension Renal disease Autoimmune disease Diabetes	Antiplatelet agent (low dose aspirin or dipyridamole) versus placebo or no antiplatelet agent	59 trials (37,560 women) with low, moderate, and high risk groups treated with or without antiplatelet agents *Outcome*: preeclampsia Secondary outcome: preterm birth and neonatal outcome	17% risk reduction with use of antiplatelet agents (RR 0.83, 95% CI 0.77, 0.89)

Trivedi 2011 [16]	7–32 weeks of gestation	*High risk criteria*: Previous severe preeclampsia Essential hypertension Underlying vascular disorder Gestational diabetes mellitus Maternal age > 40 Positive Doppler ultrasonography	Low-dose aspirin 40–160 mg versus placebo	19 trials with low risk group (16,550 women) and high risk group (11,687 women) for developement of preeclampsia Each group treated with low-dose aspirin or placebo *Outcome*: preeclampsia Secondary outcome: preterm delivery (<37 week) and IUGR	*High risk group*: preeclampsia incidence: 10.7% low-dose aspirin group, 12.5% placebo group → risk reduction of preeclampsia with low-dose aspirin: 21% (RR 0.79, 95% CI 0.65, 0.97) 16% reduction in risk for preterm delivery (RR 0.84, 95% CI 0.71, 0.99) *Low risk group*: preeclampsia incidence: 4.3% low-dose aspirin group, 4.4% placebo group → no significant risk reduction of preeclampsia wtih low-dose aspirin (RR 0.86, 95% CI 0.64, 1.17) 2% reduction in risk for preterm delivery (RR 0.98, 95% CI 0.90, 1.07)

Roberge et al. 2012 [17]	Before 16 weeks of gestation	*Risk factors*: Chronic hypertension Previous severe preeclampsia Abnormal uterine doppler Obesity First pregnancy Sjögren Syndrome	Low dose asprin 50–150 mg versus placebo	5 trials with 556 women at risk of preeclampsia treated with low dose aspirin or placebo *Outcome*: preterm and term preeclampsia	Risk reduction of preterm preeclampsia with low dose aspirin: 89% (RR 0.11, 95% CI 0.04, 0.33) No effects of low dose aspirin on term preeclampsia (RR 0.98, 95% CI 0.42, 2.33)

Villa et al. 2013 [18]	At/before 16 weeks of gestation	*Risk factors*: Abnormal uterine artery doppler flow velocimetry	Low dose aspirin 50–150 mg versus placebo/no treatment	346 women treated with aspirin or placebo *Outcome*: preeclampsia Secondary outcome: preterm (<37 week), term or severe preeclampsia	Low dose aspirin group: significant reduced risk of preeclampsia (RR 0.6, 95% CI 0.37–0.83) and severe preeclampsia (RR 0.3, 95% CI 0.11–0.69)
